# KIT (CD117) Expression in a Subset of Non-Small Cell Lung Carcinoma (NSCLC) Patients

**DOI:** 10.1371/journal.pone.0052885

**Published:** 2012-12-20

**Authors:** Albert D. Donnenberg, Ludovic Zimmerlin, Rodney J. Landreneau, James D. Luketich, Vera S. Donnenberg

**Affiliations:** 1 Hillman Cancer Center, University of Pittsburgh Cancer Institute, Pittsburgh, Pennsylvania, United States of America; 2 Department of Medicine, University of Pittsburgh School of Medicine, Pittsburgh, Pennsylvania, United States of America; 3 Department of Cardiothoracic Surgery, University of Pittsburgh School of Medicine, Pittsburgh, Pennsylvania, United States of America; Univesity of Texas Southwestern Medical Center at Dallas, United States of America

## Abstract

We have previously described the expression of CD44, CD90, CD117 and CD133 in NSCLC tumors, adjacent normal lung, and malignant pleural effusions (MPE). Here we describe the unique subset of tumors expressing CD117 (KIT), a potential therapeutic target. Tumor and adjacent tissue were collected from 58 patients. Six MPE were obtained before therapy. Tissue was paraffin embedded for immunofluorescent microscopy, disaggregated and stained for flow cytometry or cryopreserved for later culture. The effect of imatinib on CD117^high^/KIT+ tumors was determined on first passage cells; absolute cell counts and flow cytometry were readouts for drug sensitivity of cell subsets. Primary tumors divided into KIT^neg^ and KIT^+^ by immunofluorescence. By more sensitive flow cytometric analysis, CD117+ cytokeratin+ cells were detected in all tissues (1.1% of cytokeratin+ cells in normal lung, 1.29% in KIT “negative” tumors, 40.7% in KIT^+^ tumors, and 0.4% in MPE). In KIT^+^/CD117^high^, but not KIT^+^/CD117^low^ tumors, CD117 was overexpressed 3.1-fold compared to normal lung. Primary cultures of CD117^high^ tumors were sensitive to imatinib (5 µM) in short term culture. We conclude that NSCLC tumors divide into CD117^low^ and CD117^high^. Overexpression of CD117 in CD117^high^ NSCLC supports exploring KIT as a therapeutic target in this subset of patients.

## Introduction

Lung cancer accounts for 30% of male and 26% of female cancer deaths [1]. With treatment, clinical stage 1A non-small cell lung cancer (NSCLC) has a 5-year survival of 61%, while stage IV disease has a 5-year survival of only 1% [2]. NSCLC is a heterogeneous grouping of diseases, including adenocarcinomas, squamous cell carcinomas and large cell carcinomas. Identifying subsets of patients with tumors dependent on targetable pathways increases the probability of treatment success [3]. Markers associated with such subsets can be used to guide in vitro pharmacodynamic studies on primary tumor explants, which have been proposed as an approach to personalized antineoplastic therapy [4]. The present study is a followup to a survey comparing the expression of several well-known stem/progenitor markers (CD44, CD90, CD117, CD133) on cytokeratin+ cells in NSCL tumors, adjacent grossly normal lung, and NSCLC malignant pleural effusions (MPE) [5]. The rationale for the detection of stem/progenitor markers in NSCLC and normal lung is chiefly to probe for differences in expression between the clonogenic cells in these tissues in the hope of understanding their biology and identifying potential therapeutic targets [6]. The goals of the present investigation are to explore the heterogeneity of CD117 expression in NSCLC, compare CD117 expression patterns in tumor and normal lung, and determine the effect of the tyrosine kinase inhibitor imatinib on short term cultures of primary tumor tissue.

## Methods

### Patient Samples and Ethics Statement

Specimens were collected under protocols approved by the University of Pittsburgh Internal Review Board (UPCI 99–053, 020391, 0503126, 07090247). All lung cancer patients who donated tumor and/or normal lung gave written informed consent to participate in this study. Metastatic pleural effusions were exempt under the “Anonymous Use of Residual Biological Samples or Banked Tissues Samples” designation of the IRB. One sample obtained from an organ donor was approved by the University of Pittsburgh Committee for Research on the Dead. Malignant pleural effusions (MPE, 6 patients) were identified as metastatic adenocarcinoma. For solid tumors, tumor tissue (58 cases) and far-adjacent grossly tumor-free lung tissue (58 cases and 1 organ donor) were excised during port placement and were processed within 1 hour of procurement. The determination that far-adjacent tissue was tumor free was made by a pathologist or trained pathology technician by gross observation of the sample in the operating room. Samples were selected for analysis which yielded adequate cells for flow cytometric evaluation and for which formalin fixed paraffin sections were available.

### Immunofluorescent staining

Paraffin sections (5–6 µm) were prepared from embedded tissues. Tissue sections were heated (60°C, 20 min), deparaffinized (3 washes in xylenes), rehydrated by successive washes in absolute ethanol, 90% ethanol, 75% ethanol and deionized water and rinsed twice in Dako wash buffer (Dako). Antigen retrieval was performed at 125°C for 20 min in pH 9.0-EDTA buffer (Dako). After 2 washes in Dako wash buffer, the tissue sections were incubated for 1 hour in a blocking solution (PBS, 5% goat serum, 0.05% Tween 20) to reduce nonspecific antibody binding. Immunofluorescent staining was performed using the following primary antibodies: pan-cytokeratin (1∶100 (1.64 µg/mL), Dako, Cat. No. M3515, clone AE1-AE3), Ki67 (ready-to-use, Dako, Cat. No. N1633, clone MIB-1), and CD117 (1∶400 (35.7 µg/mL), Dako Cat. No. A4502, polyclonal rabbit). Primary mouse and rabbit antibodies were substituted by Dako Universal Negative Control for Mouse Antibodies (ready to use, Dako Cat. No. N1698) or Dako Universal Negative Control for Rabbit Antibodies (ready to use, Dako Cat. No. N1699), respectively. All primary antibodies and controls were incubated for overnight at 4°C. Tissue sections were washed twice using Dako Wash Buffer prior to applying biotinylated secondary goat anti-mouse (1∶500 (1.58 µg/mL), Dako Cat. No. E0433) or goat anti-rabbit antibody (1∶500 (1.52 µg/mL), Dako Cat. No. E0432) for 1 hour at room temperature. Tissue sections were washed twice with Dako wash buffer and incubated with streptavidin-Cy3 (1∶500 (2 µg/mL), Sigma, Cat. No. 6402) for 30 minutes at room temperature. Slides were washed again and tissue sections were incubated with Alexa 488-conjugated anti-pan-cytokeratin (1∶200 (2.5 µg/mL), clone AE1-AE-3, eBiosciences Cat. No. 53-9003-80) or FITC-conjugated anti-αSMA (1∶100, Sigma, Cat. No. F3777, clone 1A4) antibodies for 1 hour at room temperature. Alternatively, tissue sections (rabbit-antibody prestained slides only) were incubated with mouse anti-human pan-cytokeratin (1∶100 (1.64 µg/mL), Dako, Cat. No. M3515, clone AE1-AE3) or Ki67 (ready-to-use, Dako, Cat. No. N1633, clone MIB-1) for 1 hour at room temperature, washed twice for 5 minutes and incubated with cross-adsorbed Alexa 488-conjugated goat anti-mouse secondary antibody (1∶200 (10 µg/mL), Invitrogen, Cat. No. A11029) for 1 hour at room temperature. Stained tissue sections were washed again twice in Dako wash buffer and nuclear staining was attained through 10-min incubation with DAPI (7.15 µM Invitrogen, Cat. No. D1306). Slides were washed twice in PBS-A and mounted in Prolong Gold anti-fade reagent (Invitrogen, Cat. No. P36934). Immunofluorescent staining was observed and photographed using an epi-fluorescence microscope (Nikon Eclipse TE 2000-U).

### Single Cell Suspensions

Single cell suspensions were prepared from malignant lesions, tumor-free adjacent lung tissue, and pleural fluid [7]. Tumors and lung tissue were minced with paired scalpels and MPE were digested with type I collagenase (0.4% in RPMI 1640 medium, Cat. No. C-0130, Sigma Chemicals, St. Louis MO) and DNase (350 KU/mL, Sigma Chemicals, St. Louis MO, Cat. No. D-5025) and disaggregated through 100 mesh stainless steel screens. Viable cells were separated from erythrocytes and debris on a Ficoll-Hypaque gradient (Histopaque 1077, Sigma Chemicals). Erythrocytes were lysed using an ammonium chloride lysing solution (Beckman-Coulter, Cat No. IM3630d). A fully detailed laboratory protocol has been published as Supplementary Information (http://onlinelibrary.wiley.com/doi/10.1002/cyto.a.22156/suppinfo) [5].

### Staining and Flow Cytometry

To minimize non-specific binding of fluorochrome-conjugated antibodies, pelleted cell suspensions were pre-incubated for 5 minutes with neat decomplemented (56°C, 30 minutes) mouse serum (5 μL) [6]. Prior to intracellular cytokeratin staining, cells were stained for surface markers (2 μL each added to the cell pellet, 15–30 minutes on ice; CD44-PE (Beckman-Coulter, Cat No. A32537), CD90-biotin (BD, Cat. No. 555594), Streptavidin-ECD (Beckman Coulter, Fullerton, CA Cat. No. IM3326), CD14-PECy5 (Beckman-Coulter, Cat. No. IM2640U), CD33-PECy5 (Beckman-Coulter, Cat. No. IM2647U), Glycophorin A-PECy5 (BD Biosciences, Cat. No. 559944), CD133-APC (Miltenyi Biotech Cat. No. 130-090-854), CD117-PC7 (Beckman Coulter, Cat. No. IM3698), CD45-APCC7 (BD, Cat. No. 557833)), and fixed with 2% methanol-free formaldehyde (Polysciences, Warrington, PA). Cells were then permeabilized with 0.1% saponin (Beckman Coulter) in phosphate buffered saline with 0.5% human serum albumin (10 minutes at room temperature), cell pellets were incubated with 5 μL of neat mouse serum for 5 minutes, centrifuged and decanted. The cell pellet was disaggregated and incubated with 2 μL of anti-pan cytokeratin-FITC (Beckman Coulter, Cat. No. IM2356) for 30 minutes. Cells pellets were diluted to a concentration of 10 million cells/400 μL of staining buffer and DAPI (Sigma Chemicals, St. Louis MO, Cat. D1306 ) was added to a final concentration of 8 μg/mL [6].

Analysis was performed using the 3-laser, 9-color CyAn ADP and 10-color Gallios cytometers (Beckman Coulter, Miami FL). An effort was made to acquire a total of 10 million cells per sample at rates not exceeding 10,000 events/second. DAPI was acquired in two fluorescence channels, with PMT gain optimized for linear (cell cycle), and log (elimination of hypodiploid events) acquisition, respectively. The cytometer was calibrated to predetermined photomultiplier target channels prior to each use using SpectrAlign beads (DAKO, Cat. No. KO111) and 8-peak Rainbow Calibration Particles (Spherotech, Libertyville, IL, Cat. No. RCP-30-5A). Offline compensation and analyses were performed using VenturiOne software designed for multiparameter rare event problems (Applied Cytometry, Dinnington, Sheffield, UK). Side scatter and fluorescence parameters are displayed on a logarithmic scale. Forward scatter and DAPI fluorescence for cell cycle are displayed on linear scales. Spectral compensation matrices were calculated for each experiment using single-stained mouse IgG capture beads (Becton Dickinson, Cat. No. 552843) for each tandem antibody and hard stained beads (Calibrite, BD) for single molecule dyes (Becton Dickinson, FITC, PE (Cat. No. 349502), APC (Cat. No. 340487)).

### Flow Cytometry Analytical Strategy

The strategies used to address multiple sources of artifact encountered in disaggregated lung tissue have been described previously, including a detailed standard operating procedure included as an online supplement [5]. Mechanical and enzymatic digestion, required for flow cytometry of solid tissues, has the potential to introduce significant artifact. In addition to tumorigenic cells, tumor samples contain stromal cells, reactive cells and immune cells [6], [8], [9]. The gating strategy used for this investigation is provided in supplementary figure S1, and the analysis of stem/progenitor markers (CD44, CD90, CD117, CD133) and DNA content, in KIT+ and KIT- tumors, was confined to the cytokeratin positive epithelial component of both tumor and adjacent tissues [7], [10], [11]. Following this strategy shown in supplementary figure S1, the denominator for all determinations in this study was non-hematopoietic, non-mesothelial [12], singlet cells with at least 2N DNA. Examples of complete multiparameter analyses of representative normal lung, KIT+ and KIT- tumors, and a metastatic pleural effusion are presented in supplementary figure S2. For each analytical region, event number and mean fluorescence intensity were exported to the SYSTAT (Chicago, IL) for exploratory analysis, graphics and statistical analysis.

### Cryopreservation of Single Cell Suspensions

Cells were resuspended to a maximum concentration of 50×10^6^/mL in DMEM medium (Invitrogen/GIBCO, Grand Island, NY, Cat. No. 11965) containing 40% newborn calf serum and chilled on wet ice. An equal volume ice-cold of cryoprotective medium (20% dimethylsulfoxide (Sigma, Cat No. D5879) in DMEM medium) was added and the cells aliquoted to freezing vials (Sarstedt, Newton NC Cat. No. 72.694.10) prechilled in a freezing rack (Stratagene, Santa Clara CA) and transferred to a −80°C freezer for at least 12 hours, after which they were transferred to a liquid nitrogen freezing vessel.

### In Vitro Culture Conditions for the Growth of Primary Lung Epithelium and Tumor-derived Epithelial Cells

Single cell suspensions prepared for each tissue were cultured in modified Bronchial Epithelial Cell Growth Medium (BEGM, Lonza, Walkersville, MD Cat. No. CC-3170 BEGM BulletKit (CC-3171 & CC-4175)) with all supplements and 10% of cell-free clarified pleural fluid at a density of 50,000 to 500,000 cells/cm^2^ for initial expansion and 10,000 cells/cm^2^ for growth and drug treatment. Individual cultures were scored for growth and photographed for the presence of colonies, as well as cellular and colony morphologies. Confluent wells were trypsinized, split 1∶3 and used for drug exposure and harvested for flow cytometry and cytospins.

### Imatinib Treatment of Primary and Metastatic Tumor-derived Epithelial Cells

Expanded primary cell cultures (passage 1) were seeded at 10,000 cells/cm^2^. Imatinib, 5 μM final concentration [13], [14], [15] (LC Laboratories, Cat.No. I-5508, MW 589.71) or vehicle control was added to duplicate cultures. After 4 days incubation at 37°C, 5% CO_2_, saturated humidity, cells were removed with trypsin (Lonza, 0.05%, 5 min at 37°C) and washed, manually counted (hemocytometer, trypan blue viability). Cytocentrifuge preparations were made on single cell suspension and stained with Wright-Giemsa stain. Single cell suspensions were stained for flow cytometry as described above. Measured parameters were: intracellular pan-cytokeratin, CD90, CD44, EpCAM, CD45, hematopoietic lineage cocktail and DAPI, using reagents as described above.

### Statistical Analysis

Statistical analyses were performed using SYSTAT version 13. SYSTAT was also used to create box plots. Cytometry data were tested for normality and log transformed prior to analysis when required to meet assumptions of normality. Student's 2-tailed t-test was used to compare cell frequencies between 2 groups; ANOVA was used to compare multiple groups (Tukey-Kramer honestly significant difference test for contrasts). Student's paired t-test (2-tailed) was used to compare cells cultured in the absence or presence of imatinib. Raw survival analysis (death from all causes) was performed using the Kaplan-Meier estimator and the log-rank test.

## Results

### Immunofluorescent and flow cytometric detection of stem/progenitor markers in non-small cell lung tumor and adjacent tumor-free tissue

Flow cytometry permits analysis of multiple markers on single cells but loses histological context and is subject to selection artifact during sample disaggregation. To understand multidimensional flow cytometric data in context, we examined key markers to be included in our flow panel by immunofluorescence microscopy. Formalin fixed paraffin embedded sections were prepared from 20 NSCLC tumors, 1 benign lung tumor, and 17 paired adjacent grossly normal lung samples (NL) plus 1 normal lung procured from an organ donor (NL). Epithelial cells were identified as cytokeratin+. Sections were also stained for CD117, α-smooth muscle actin (αSMA) and the proliferation marker, Ki-67.

**Figure 1:**
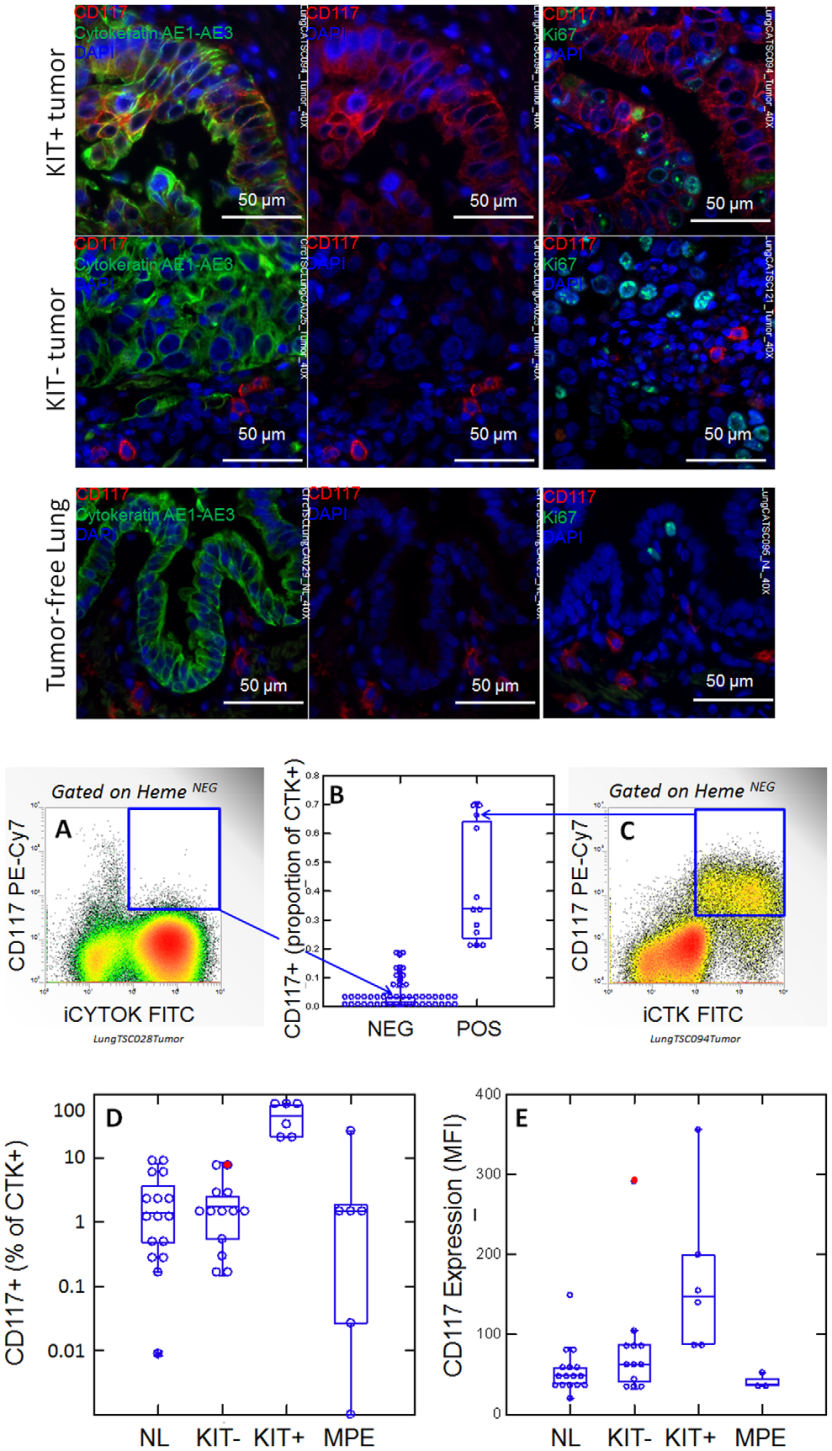
CD117 expression in normal lung (NL) and NSCLC. Photomicrographs: Expression of CD117 and Ki-67 in NSC lung cancer and normal lung. The left columns show sections stained with CD117 (red), cytokeratin (green) and DAPI (blue). Sections in the center column show CD117 (red) and DAPI (blue) only, in order to reveal CD117 staining obscured by bright cytokeratin expression. Sections in the right column shows CD117 (red), the proliferation marker Ki67 (green) and DAPI (blue). Tumors were classified as KIT+ or negative on the basis of CD117 immunofluorescent staining of FFPE. In KIT+ tumors (top photomicrographs) CD117 (red stain, center and right panels) was expressed in virtually all cytokeratin+ tumor cells (green stain, left panels). Ki-67+ proliferating cells (green stain, right panels) were frequently seen among CD117+ tumor cells. In KIT negative tumors (center row of photomicrographs), only solitary CD117+ mast cells were detected (red stain, center and right panels). Proliferating Ki-67+ cells were frequent among cytokeratin+ CD117 negative tumor cells. Normal tumor-adjacent lung also appeared to lack CD117 expression among cytokeratin+ airway cells (bottom photomicrographs). Proliferating Ki-67+ cells were infrequent and confined to the basal layer of airway epithelium. When all NSCLC tumors are considered together, flow cytometry revealed bimodal CD117 expression (center panels A-C). Cells were gated on hematopoietic lineage negative singlet events with DNA content ≥2N, as detailed in supplementary figure 1. The percent of CD117+ cells, among cytokeratin positive cells, plotted on a linear scale, allowed the distinction between CD117low (≤20% positive) and CD117high (>21%). An example of CD117 detection on a tumor single cell suspension is given for a KIT negative tumor (panel A) and a KIT+ tumor (panel C). Arrows indicate the corresponding data points (open circles). The percent CD117+ cells among cytokeratin+ cells, shown on a log scale, reveals the presence of detectable CD117+/cytokeratin+ cells in normal lung, KIT “negative” tumors, and malignant pleural effusions (MPE, panel D). The proportion of cells expressing CD117 in KIT+ tumors was statistically significant from that of normal lung (NL, p = 0.004), KIT negative tumor (p = 0.008) and MPE (p = 0.001). Panel E shows the relative intensity of CD117 expression on CD117+ cells, as measured by CD117 mean fluorescence intensity (MFI). In KIT+ tumors, CD117 expression on CD117+ cells was significantly greater than that of normal lung (NL, p = 0.002), KIT negative tumor (p = 0.026) and malignant pleural effusion (MPE, p = 0.002). The filled red circle indicates that the sole outlier in CD117 expression in the KIT negative group, is at the top of the range of percent CD117+. Box plots (panels B, D, E) indicate the sample median and quartiles, the whiskers indicate the sample range, exclusive of outliers.

Figure 1 shows photomicrographs detailing expression of cytokeratin, CD117 and the proliferation marker Ki-67. NSCLC segregated into CD117+ (KIT+) and CD117- tumors (KIT-) by immunofluorescence. In KIT+ tumors, CD117 was expressed in the majority of cytokeratin+ tumor cells. Ki-67+ proliferating cells were frequently seen among CD117+ tumor cells. In KIT negative tumors, CD117 was expressed on scattered cytokeratin negative mast cells, which are prominent in remodeling lung disease [16]. The presence of CD117 on mast cells serves as an internal positive control for immunofluorescent detection of KIT, which can be problematic in FFPE tissues [17]. Proliferating Ki-67+ cells were frequent among cytokeratin+ tumor cells. CD117 was not detected among cytokeratin+ airway cells by immunofluorescence in tumor-free adjacent lung, and proliferating Ki-67+ cells were infrequent.

CD117 expression was quantified on cytokeratin+ cells by flow cytometry in 58 NSCLC patients 13 of whom were studied with both immunofluorescence and high resolution flow cytometry (simultaneous detection of cytokeratin, DNA, CD44, CD90, CD117, CD133, and hematopoietic cells) (Figure 1, panels A–E). By flow cytometry, which is more sensitive and quantitative than immunofluorescence, CD117 expression on cytokeratin+ cells was clearly bimodal, with 13 tumors (22.4%) having >20% CD117+ cells (Figure, panels A-C). The flow cytometric grouping (low, high) was entirely concordant with immunohistochemistry (negative, positive) in the 13 patients studied by both methods. In KIT positive tumors, a geometric mean of 40.7% (22.7, 73.1%, lower [LCI] and upper [UCI] 95% confidence intervals) of cytokeratin+ cells were CD117+, compared to 1.29% (0.59, 2.8%) in KIT “negative” tumors (Figure 1D). This was comparable to the frequency of CD117+ cells among cytokeratin+ cells of tumor-free lung (1.1%, LCI95 = 0.4%, UCI95 = 2.8%). CD117 was also expressed in a small but variable proportion of cytokeratin+ MPE cells (0.4%, LCI95 = 0.01%, UCI95 = 17.5%). CD117 expression on cytokeratin+ cells can be quantified in two ways: 1) as the proportion of cytokeratin+ cells bearing detectable CD117 (*i.e.* percent positive), or 2) as the expression level (*i.e.* the relative number of CD117 molecules per cell on CD117+ cells). Quantification of CD117 relative expression level among cytokeratin+/CD117+ cells, as measured by mean fluorescence intensity of CD117 staining, showed a 3.1-fold increase in expression in KIT+ tumors compared to NL (p = 0.002, Figure 1E). The sole high CD117 expressor among the KIT “negative” tumor group was at the upper range of percent CD117+, suggesting transition or misclassification (Figure 1, D and E, red circles). The level of CD117 expression on cytokeratin+/CD117+ cells was statistically indistinguishable between NL, KIT “negative” tumor and MPE (Figure 1E).

The results of immunofluorescent staining (cytokeratin, CD117, αSMA and Ki-67) of 20 NSCLC tumors, 1 benign tumor, and 17 paired adjacent grossly normal lung samples plus 1 normal lung are summarized in Tables 1 and 2. Analysis was divided into markers expressed on the parenchymal component and markers expressed in the airways or epithelial tumor component (as defined by cytokeratin expression). All 20 tumor sections evidenced areas of malignant epithelial (cytokeratin+) cells interspersed with stromal cells, blood vessels and inflammatory cells. Small airways were visualized in all samples of tumor-adjacent NL.

**Table 1:** Immunofluorescent staining of 21 lung masses.

				Parenchyma			Epithelium			
Experiment	TNM	Stage	Tumor type	αSMA	Ctk	CD117	αSMA	Ctk	CD117	Ki67
LungCATSC084			Benign (granulomatous)	+ (S,V)	−	+ (M)	−	+	−	+
LungCATSC138	T3N2MX	IIIA	Adenocarcinoma	+ (S,V)	−	−	−	+	−	+
CircTSCLungCA023	T1N1MX	IIA	Squamous Cell Carcinoma	+ (S,V)	−	+ (M)	−	+	−	+
CircTSCLungCA024	T1N1MX	IIA	Squamous Cell Carcinoma	+ (S,V)	−	-	−	+	−	+
CircTSCLungCA025	T1N1MX	IIA	Adenocarcinoma	+ (S,V)	−	+ (M)	−	+	neg to dim	+
CircTSCLungCA028	T2N0MX	IB	Adenocarcinoma	+ (S,V)	−	-	−	+	−	+
LungCATSC081	T1N1MX	IIA	Large Cell Carcinoma	+ (S,V)	−	-	−	+	−	+
LungCATSC083	T1N0MX	IA	Squamous Cell Carcinoma	+ (S,V)	−	+ (M)	−	+	−	+
LungCATSC087	T1N2MX	IIIA	Pleomorphic Carcinoma	+ (S,V)	−	+ (M)	−	+	−	+
LungCATSC095	T2N0MX	IB	Squamous Cell Carcinoma	+ (S,V)	−	+ (M)	−	+	−	+
LungCATSC121	T1N0MX	IA	Invasive Adenocarcinoma	+ (S,V)	−	+ (M)	−	+	neg w/sparse dim	+
LungCATSC135	T1N2MX	IIIA	Invasive Adenocarcinoma	+ (S,V)	−	-	−	+	−	+
LungCATSC136	T3N0MX	IIB	Invasive Squamous Carcinoma	+ (S,V)	−	+ (M)	−	+	−	+
CircTSCLungCA029	T2N0MX	IB	Neuroendocrine Carcinoma	+ (S,V)	−	+ (M)	−	+	+	+
LungCATSC072	T3N0MX	IIB	Invasive Adenocarcinoma	+ (S,V)	−	+ (M)	−	+	+	+
LungCATSC074	T2N0MX	IB	Basaloid Squamous Cell Carcinoma	+ (S,V)	−	-	−	+	+	+
LungCATSC089	T1N0MX	IA	Adenocarcinoma	+ (S,V)	−	+ (M)	−	+	+	+
LungCATSC093	T1N0MX	IA	Adenocarcinoma	+ (S,V)	−	+ (M)	−	+	+	+
LungCATSC094	T3N0MX	IIB	Adenocarcinoma	+ (S,V)	−	+ (M)	−	+	+	+
LungCATSC098	T1N0MX	IA	Invasive Adenocarcinoma	+ (S,V)	−	+ (M)	−	+	+	+
LungCATSC137	T2N0MX	IB	Invasive Lepidic Adenocarcinoma	+ (S,V)	−	+ (M)	−	+	+	+

Expression of alpha smooth muscle actin (αSMA), cytokeratin, CD117 and the proliferation marker Ki67 was assessed in parenchymal (cytokeratin−) and epithelial (cytokeratin+) tissues. S =  stroma, H =  hematopoietic lineage, V =  blood vessels, M =  mast cells.

**Table 2:** Immunofluorescent staining of 18 tumor free lung samples.

			Parenchyma			Epithelium		
Exp	Histology	Inflammatory infiltrate	αSMA	Cytokeratin	CD117	αSMA	Cytokeratin	CD117
LungCATSC084	Adjacent	−	+	−	mast	−	+	−
CircTSCLungCA023	Adjacent	−	+	−	mast	−	+	−
CircTSCLungCA024	Adjacent	−	+	−	mast	−	+	−
CircTSCLungCA025	Adjacent	−	+	−	mast	−	+	−
CircTSCLungCA028	Adjacent	−	+	−	mast	−	+	−
LungCATSC081	Adjacent	−	+	−	mast	−	+	−
LungCATSC083	Adjacent	−	+	−	mast	−	+	−
LungCATSC087	Adjacent	−	+	−	mast	−	+	−
LungCATSC095	Adjacent	−	+	−	mast	−	+	−
LungCATSC121	Adjacent	−	+	−	mast	−	+	−
CircTSCLungCA029	Adjacent	+	+	−	mast	−	+	−
LungCATSC072	Adjacent	+	+	−	mast	−	+	−
LungCATSC074	Adjacent	−	+	−	mast	−	+	−
LungCATSC089	Adjacent	−	+	−	NA	−	+	NA
LungCATSC093	Adjacent	−	+	−	NA	−	+	NA
LungCATSC094	Adjacent	−	+	−	mast	−	+	−
LungCATSC098	Adjacent	−	+	−	mast	−	+	rare
NL005	Organ donor	−	+	−	NA	−	+	NA

Expression of alpha smooth muscle actin (αSMA), cytokeratin and CD117 was assessed in parenchymal (cytokeratin−) and epithelial (cytokeratin+) tissues. NA  =  not available. Seventeen samples were from tumor adjacent tissue, 1 sample was from a heart-beating cadaveric organ donor.

CD117+ mast cells were present in 14/20 tumors, always visualized as cytokeratin negative solitary cells. Tumors divided into CD117+ (10/20) and CD117- (10/20). In CD117+ tumors, CD117 was expressed in the majority of cytokeratin+ tumor cells, and was associated with a glandular morphology in 9/10. The epithelium in the benign lung mass was proliferative but CD117 was undetectable.

Two of 17 resected tumor-free lung samples evidenced gross inflammatory infiltrates; all samples had cytokeratin negative CD117+ mast cells surrounding airways. CD117 was not detected on cytokeratin+ airway epithelial cells, with the exception of rare single cells in one sample. Ki67+ proliferating cells were rare but present in all airways.

### Co-expression of CD44, CD90 and CD133 on CD117+ cytokeratin+ cells.

Several markers have been used to identify stem and progenitor cells in solid tissues and tumors. Among these are CD44, CD90, CD117 and CD133. As demonstrated in Figure 1, NSCLC tumors could be grouped into CD117 low (KIT “negative”) and high (KIT+) when assayed by flow cytometry.

**Figure 2:**
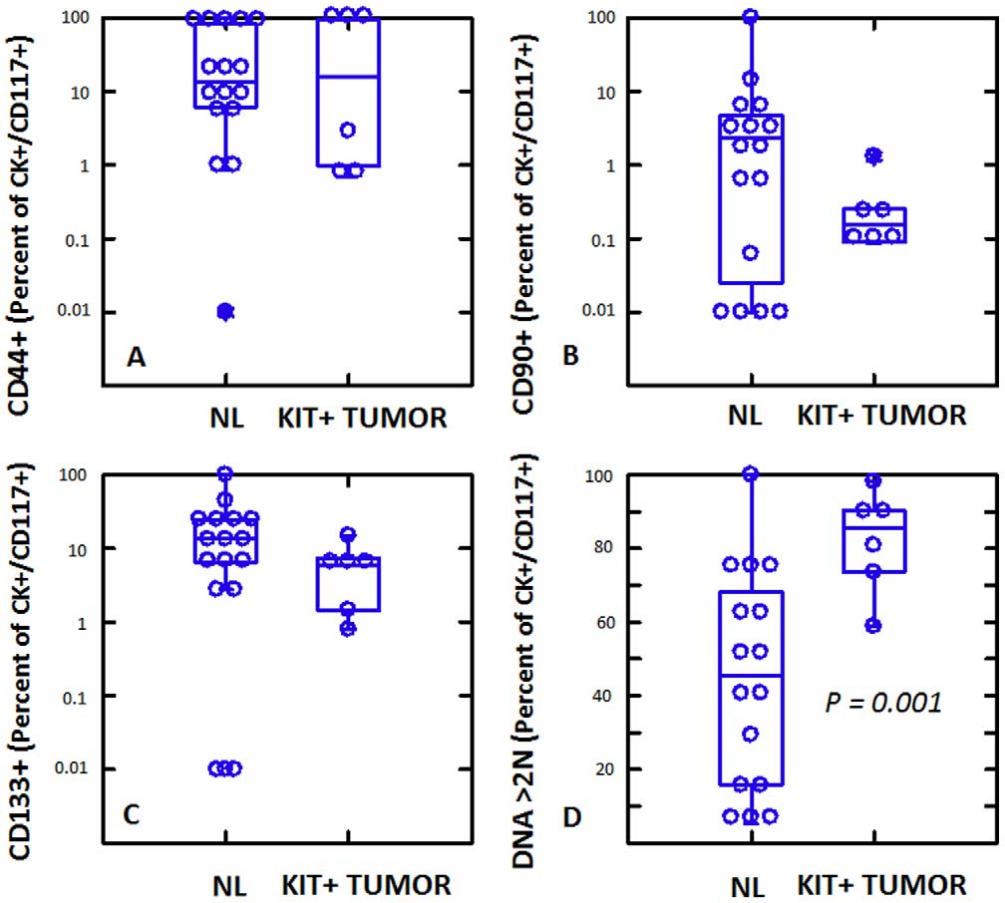
High resolution phenotype of cytokeratin+ CD117+ cells from normal lung (NL) and CD117+ tumors. Cytokeratin+/CD117+ cells were identified and expression of CD44, CD90, CD133 and DNA content was determined. With the exception of DNA content, which was significantly higher on CD117+ tumor cells (Student's 2-tailed t-test), CD117+ cells in normal lung and tumor were indistinguishable.

In KIT+ NSCLC, expression of CD117 provides a potential link between a large proportion (44%) of tumor cells and their less prevalent normal lung counterpart, the multipotent lung stem cell [18]. In order to investigate this correlation further, we quantified expression of the stem/progenitor associated markers CD44, CD90 and CD133 on the CD117+/cytokeratin+ population from KIT+ NSCLC and normal lung (Figure 2, supplementary Figure 2). CD44+ and CD133+ cells comprised substantial subpopulations (median 5.8–16.1%) of CD117+/cytokeratin+ cells in both tumor and normal samples (Figure 2 A,C), whereas CD90+ cells were less prevalent (median 0.2–2.3%, Figure 2B). Notably, expression of these three stem/progenitor associated markers on normal CD117+ lung stem cells was statistically indistinguishable from their expression on CD117+ tumor cells (analysis of variance, Figure 2A–C). Normal and tumor CD117+/cytokeratin+ cells were distinguished only by DNA content, where tumor had a significantly higher proportion of cells with >2N DNA (Figure 2D, p = 0.001, Student's 2-tailed t-test).

### Stem/progenitor marker expression and disease progression

**Figure 3:**
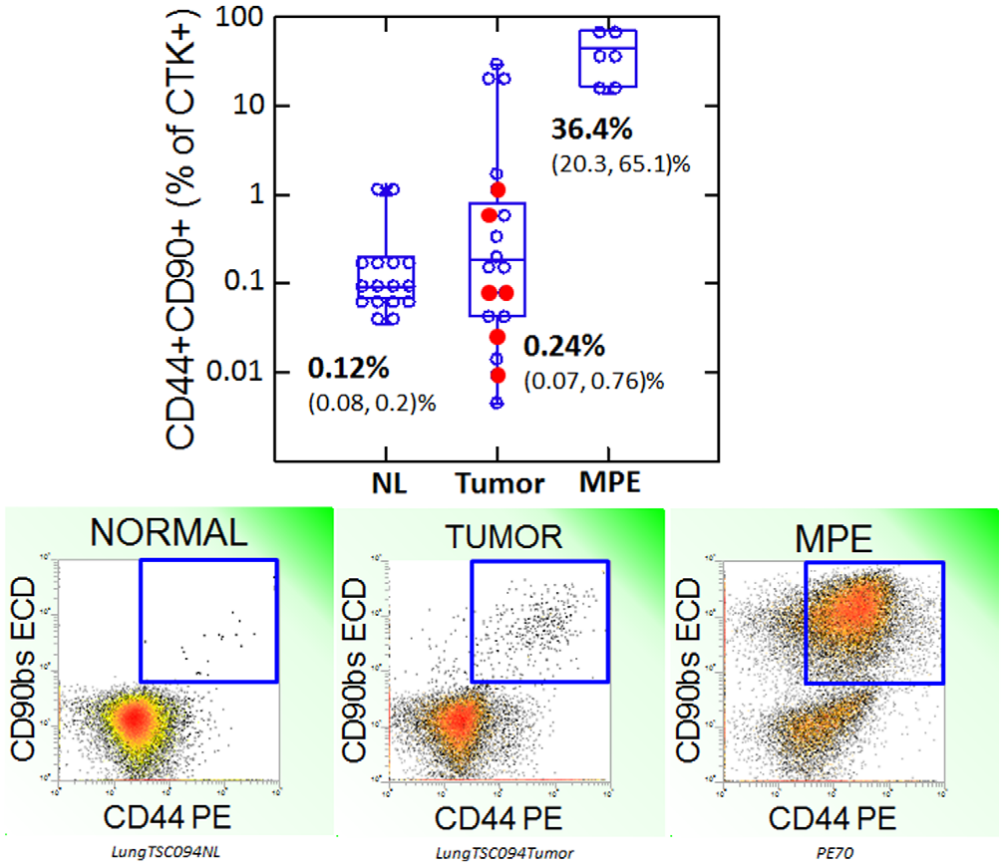
Coexpression of CD44 and CD90 as a marker of progression. Top panel: Box plot of the proportion of cytokeratin+ cells coexpressing CD44 and CD90. CD44+/CD90+ cytokeratin+ cells are rare in normal lung, heterogeneously distributed in NSCL tumors and prevalent in NSCLC malignant pleural effusions. KIT+ tumors are shown as filled circles. The mean percent positive (lower, upper 95% confidence interval) is shown for each sample type. Bottom panels: Examples of flow cytometric detection of CD90/CD44 coexpression on cytokeratin+ adjacent normal lung, NSCL tumor and NSCLC malignant pleural effusion.

Evaluation of stem/progenitor marker expression on cytokeratin+ cells from normal lung, untreated KIT+ and KIT- primary NSCLC, and recurrent metastatic disease presents the opportunity to correlate the presence of these markers with disease progression. The frequency of CD44+/CD90+/cytokeratin+ cells increased progressively from normal lung, to primary lung tumor to MPE (Figure 3). The frequency of CD44+/CD90+/cytokeratin+ cells did not differ in KIT+ (Figure 3, filled circles) and KIT negative tumors. However, the frequency of CD44+/90+/cytokeratin+ cells was greatly increased in MPE (p = 0.000), in contrast to the proportion of CD117+ cells which actually decreased in MPE to levels comparable to that of normal lung (Figure 1). Neither CD44/CD90 expression, nor CD117 grouping (high, low) were predictive of overall survival (50% survival at 5.2 years, mean follow-up  = 4.3 years). The frequency of CD133+ cells among cytokeratin+ cells was statistically indistinguishable in normal lung, primary tumor and metastatic tumor (supplementary Figure S3).

### Imatinib sensitivity of NSCLC in vitro

**Figure 4:**
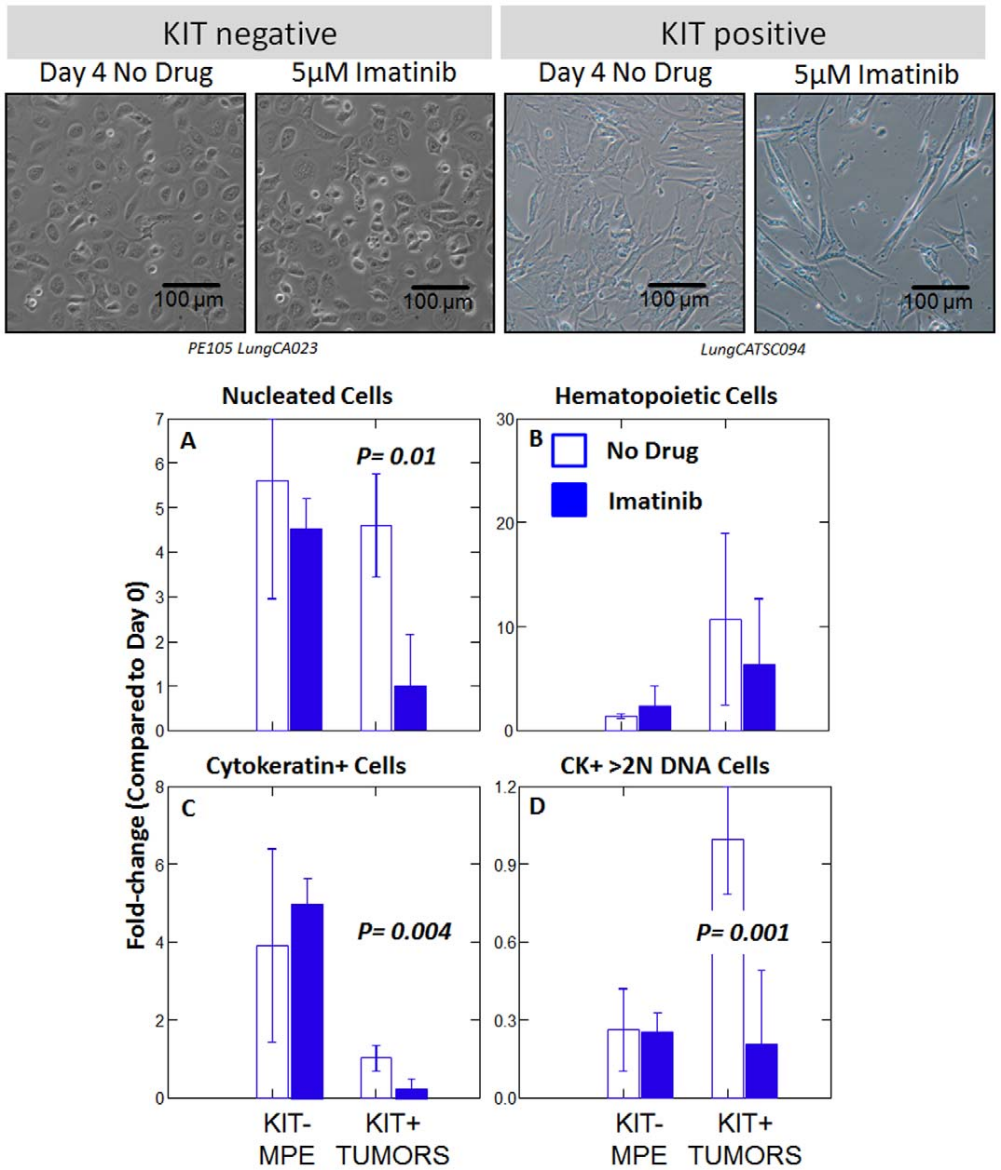
Effect of imatinib on the growth of three KIT+ lung tumors and a KIT negative malignant pleural effusion (MPE). First passage cells derived from primary patient material was incubated in the presence or absence of imatinib (5 µM) for 4 days. Top panels, photomicrographs of cultured cells. Histograms show the fold-change in cell number for specific cell subsets, defined by flow cytometry, in the absence or presence of drug. Bars show mean values. Error bars show the standard error of the mean. The p values were determined by Student's paired t-test (2-tailed). Supplementary figure S4 shows the gating strategy used to define the populations of interest.

Primary tumor isolates were disaggregated and single cell suspensions were cryopreserved in LN_2_. Three CD117+ tumors and one CD117 negative malignant pleural effusion (MPE) were thawed, passaged once, trypsinized, plated in the presence or absence of 5 μM imatinib, and cultured for 4 days, after which time they were trypsinized, counted and stained for flow cytometry (Figure 4, supplementary Figure 4). DNA content (DAPI staining), and cytokeratin expression were measured. CD117 expression was not evaluated after culture because it proved to be trypsin sensitive (data not shown). Absolute cell numbers were calculated by multiplying total viable cell counts by the proportion of cells in a given analytical region. Data are expressed for each subpopulation as fold-change in cell number compared to day 0 (Figure 4, panels A–D).

Imatinib did not exert a detectable effect in the single KIT negative MPE tested. In primary KIT+ tumor cultures, nucleated tumor cells expanded in the absence (4.6±0.4-fold), but not the presence (1.2±0.5-fold) of imatinib (p = 0.01, Figure 4A). Passenger cells of the hematopoietic lineage expanded in the absence (14.8±4.8-fold, mean ± standard error) and presence (6.2±2.3-fold) of drug (Figure 4B). The cytokeratin+ tumor population, as a whole, did not expand in 4-day culture in the absence of drug (1.0±0.1-fold, Figure 4C), but addition of imatinib caused a marked decrease in cell number (0.2±0.1-fold, p = 0.004). Among the cytokeratin+ aneuploid population (>2N DNA, Figure 4D, KIT+ tumors) in which no net growth was observed (1.0±0.1-fold), addition of imatinib resulted in a significant decrement (0.2±0.1-fold, p = 0.001) in cell number.

DNA content analysis of the cytokeratin+ CD44+/CD90+ tumor population, a subset shown to be highly tumorigenic in other epithelial cancers [6], [11], [19], revealed an imatinib-mediated right-shift in DNA profile consistent with the expansion of early S-phase cells and cell cycle arrest (supplementary Figure S4, bottom right). Consistent with this observation imatinib did not affect the cell cycle profile of cultured MPE (not shown).

## Discussion

With the advent of targeted therapies such as imatinib in chronic myeloid leukemia (CML) [20], [21], [22] and trastuzumab in breast cancer [23], there has been an effort to identify subsets of cancer patients with tumors reliant on pathways that can be disrupted without intolerable collateral damage to the cells responsible for tissue maintenance and regeneration. The type III receptor tyrosine kinase KIT is a highly conserved signaling protein involved in the regulation of apoptosis, proliferation, differentiation and adhesion [17]. When activated by dimerization through ligation of its extracellular domain, it exerts different biological effects depending on the tissue in which it is expressed. Agents targeting tyrosine kinase activity have an excellent therapeutic index, effectively targeting constitutively activated *bcr-abl* in CML and *KIT* in gastrointestinal stromal tumors (GIST) [24], [25], [26], with minimal toxicity compared to cytotoxic therapies [27]. Because imatinib also targets wild-type KIT [28], which is expressed on a subset of hematopoietic stem/progenitor cells [29], cytopenias are a common finding [30]. Despite the efficacy of imatinib in GIST with imatinib-sensitive activating *KIT* mutations, tyrosine kinase inhibitors have been disappointing in small cell carcinoma of the lung (SCCL) [31], [32], [33], 28-88% of which are reported to be KIT+ [17]. Notably *KIT* appears to be wild-type in SCCL [34], rendering it at least theoretically sensitive to imatinib. One interpretation of these data is that expression of a pathway normally involved in cell growth and survival can only be a target of therapy if it is constitutively active and if the tumor depends on it *uniquely* for its dysregulated growth. In CML and GIST, translocation of the *abl* TK or activating *KIT* mutations not only provide mechanisms for upregulated proliferation and apoptosis resistance, they may also render the tumors entirely dependent on type III TK activity, and therefore susceptible to its inhibition. In normal tissues expressing type III TKs, redundant mechanisms may explain why the toxicity of TK inhibitors is limited rather than severe (*e.g.* most imatinib associated cytopenias).

The data presented in the present study demonstrates that NSCLC tumors, like SCCL can be subsetted on the basis of KIT expression (Figure 1). Immunofluorescence studies on FFPE tumor confirm the works of other [35], [36], [37], dividing tumors into KIT+ and KIT negative. However, our far more sensitive flow cytometric determinations demonstrate for the first time that KIT+/cytokeratin+ cells are present in all NSCLC tumors, with CD117^high^ tumors accounting for about 22% of cases. We have also shown that tumors classified as KIT+ by immunofluorescence (and CD117^high^ by flow cytometry) not only have a higher proportion of KIT+ cells than those deemed “negative”, each KIT+ cell has a 2.7-fold higher expression of CD117 compared to KIT “negative” tumors and a 3.1-fold increase compared to normal lung (Figure 1), a determination that was not possible in previous reports relying on immunohistochemistry alone. Comparing the prevalence and relative expression of CD117 on tumor and normal lung is particularly relevant, since CD117 expression has recently been used as a criterion to sort-purify human lung stem cells capable of giving rise to airways and alveoli [18]. Absence of pulmonary toxicity in patients receiving imatinib or other type III TK inhibitors suggests that KIT activity is not critical to the function of normal lung stem or progenitor cells. In contrast, the increased CD117 receptor density that we demonstrated on KIT+ NSCLC could conceivably result in cross-phosphorylation and constitutive KIT activity, rendering KIT+ NSCLC tumors dependent on KIT expression and susceptible to its inhibition. The crucial issue appears to be whether tumors *depend* on KIT activity for their survival or growth, and not whether tumor cells merely express KIT. A possible clue comes from the findings of Herpel et al. [37], who demonstrated a trend toward worse overall survival in a large series of resectable NSCLC patients in the subgroup of patients with relapse whose primary tumor was KIT+ and >3 cm (hazard ratio  = 2.6, p = 0.08).

Despite the fact that KIT+ NSCLC tumors have an increased prevalence, and increased expression of CD117, as well as an increased proportion of cycling/aneuploid cells, detailed subset analysis of the stem/progenitor markers CD44, CD90 and CD133 on CD117+/cytokeratin+ tumor cells revealed no differences between the tumor cells and CD117+ normal lung stem cells (Figure 2). As in normal lung stem cells, CD44 tended to coexpress with CD117, but CD90 was largely independent of CD117 expression. CD133 was expressed on a comparable subset of CD117+/cytokeratin+ tumor and normal cells.

In cytokeratin negative cells, coexpression of CD44 [38] and CD90 [39] is consistent with the phenotype of mesenchymal stem cells. In the breast, cells positive for the epithelial lineage marker cytokeratin, CD90 and CD44 may identify epithelial ductal progenitor cells as well as clonogenic tumor cells [11]. This has also been demonstrated in liver cancer [19], [40]. Our data are consistent with the idea that in NSCLC, the prevalence of CD44+/CD90+/cytokeratin+ cells, which are present but rare in normal lung, is associated with metastasis and a potential increase in the frequency of tumorigenic cells. The prevalence of CD44+/CD90+/cytokeratin+ cells in primary tumor was intermediate between normal lung and MPE, with a distinct cluster of 3 high prevalence tumors (Figure 3). CD117+ tumors spanned the median for CD44/CD90 expression. The coexpression of CD44+/CD90+/cytokeratin+ cells in normal lung is of unknown significance, as these cells appear to be distinct from the majority of CD117+ cells.

In vitro sensitivity assays of imatinib activity have relied exclusively on cancer cell lines [41], [42], [43], [44], [45]. In SCLC in vitro responsiveness has not correlated with clinical activity. In the present study, we evaluated imatinib sensitivity using first-passage clinical isolates as our indicator cells and detailed flow cytometric analysis to measure the drug effects on these heterogeneous explants after short-term culture. Addition of imatinib to the cultures resulted in a statistically significant decrease in the total number of cells, the number of cytokeratin+ cells, and the number of cells with >2N DNA (Figure 4). In the presence of drug, DNA profiles were consistent with cell cycle arrest in S-phase (supplementary Figure S4), a known mechanism of action of type III TK inhibitors [46], [47]. In contrast, a single cultured KIT “negative” MPE was unaffected by the addition of imatinib. Our data in clinical isolates are consistent with the findings of Levina et al., who demonstrated imatinib sensitivity of “cancer stem cells” expanded from spheroids derived from the lung cancer cell lines H460 and A549, and demonstrated the presence of an autocrine loop in which KIT + tumor cells secret the kit-ligand Stem Cell factor [45]. Taken together, our data have identified subsets of NSCLC patients with increased prevalence and expression of the type III TK, KIT, compared to KIT “negative” (CD117^low^) NSCLC and normal lung. The in vitro imatinib sensitivity of primary cultures of KIT+ tumors, and the known limited toxicities of type III TK inhibitors, warrants further work examining the mechanisms leading to KIT overexpression, the possibility of constitutive KIT activation, and the effect of imatinib on downstream targets in the KIT pathway.

## Supporting Information

Figure S1 **Gating strategy.** The gating strategy used to remove cell clusters, debris, red and white blood cells and mesothelial cells is shown. A freshly excised non-small cell lung carcinoma was disaggregated and prepared for flow cytometry as described. 4.9 million events were acquired. Region percents indicate mean values (lower and upper 95% confidence intervals) for 21 NSCLC tumors. Top panels (left to right): Forward light scatter pulse analysis is used to eliminate cell clusters and retain singlet cells (A); the DNA stain DAPI is used to define nucleated cells with ≥2N DNA (B); Forward versus side light scatter is shown for total nucleated cells; CD45 versus a cocktail of CD14, CD33 and glycophorin A is used to gate out hematopoietic (heme) cells; CD14+ is also expressed on mesothelial cells which may be present in pleural effusions; Tissue lymphocytes, identified as CD45bright, are used as an internal standard for 2N DNA content and low light scatter morphology. Bottom panels (left to right): Detection of cytokeratin+ cells on non-heme singlets with ≥2N DNA; enumeration of small (lymphoid light scatter) cells among cytokeratin+ cells; DNA content of cytokeratin+ cells (2N and >2N). This gating strategy was used for all subsequent analyses.
(TIF)

Figure S2 **Flow cytometric detection of stem/progenitor markers on cytokeratin+ cells.** The gating strategy described in supplementary figure S1 was used to eliminate sources of artifact and identify cytokeratin+ cells. To identify cycling/aneuploid cells, cytokeratin+ cells were divided into those with 2N DNA and those with >2N DNA (Figure S1). Representative analyses are shown for normal lung, KIT positive and negative tumors, and a metastatic pleural effusion. The numbers shown in the analytical regions represent the geometric means (percent of 2N or >2N cytokeratin+ cells) of determinations made on all samples. Parentheses enclose the lower and upper 95% confidence intervals about the geometric means.
(TIF)

Figure S3 **Expression of CD133 on cytokeratin+ cells.**
(TIF)

Figure S4 **Effect of imatinib on primary lung cancer epithelial cultures.** First passage tumor cell isolates were cultured for 4 days in the absence or presence of imatinib (5 μM). Cells were gently trypsinized, counted and analyzed by multi-parameter flow cytometry. Three individual CD117+ tumor preparations were analyzed in duplicate. Photomicrographs and histograms are from a single representative experiment. Event numbers and fold-changes were calculated from the pooled data of 3 independent experiments (mean ± standard error). Singlet cells with ≥2N DNA (first histogram column) increased 4.6-fold in culture, but did not significantly proliferate in the presence of drug. CD45-/heme-lineage- cells also increased 4-fold in culture in the absence, but not the presence of drug, while CD45+ inflammatory cells increased in number in the absence (14.8-fold) or presence (6.2-fold) of drug. Although imatinib caused stasis of non-hematologic cytokeratin- stromal cells, it actually decreased the number cytokeratin+ cells 0.2-fold) and its CD90+/CD44+ subset, a phenotype associated with tumor stem/progenitor cells. The broadening of the cell cycle profile in the presence of imatinib (last column, bottom row) is consistent with drug-mediated cell cycle arrest in S-phase. The bar indicates cells with >2N DNA content (compared to an internal control: resting CD45+ lymphocytes).
(TIF)
