# Supplementary figures and images for: KIT (CD117) Expression in a Subset of Non-Small Cell Lung Carcinoma (NSCLC) Patients
Source: PLoS One. 2012 Dec 20;7(12):e52885. doi: 10.1371/journal.pone.0052885 (PMC3527622; doi:10.1371/journal.pone.0052885)

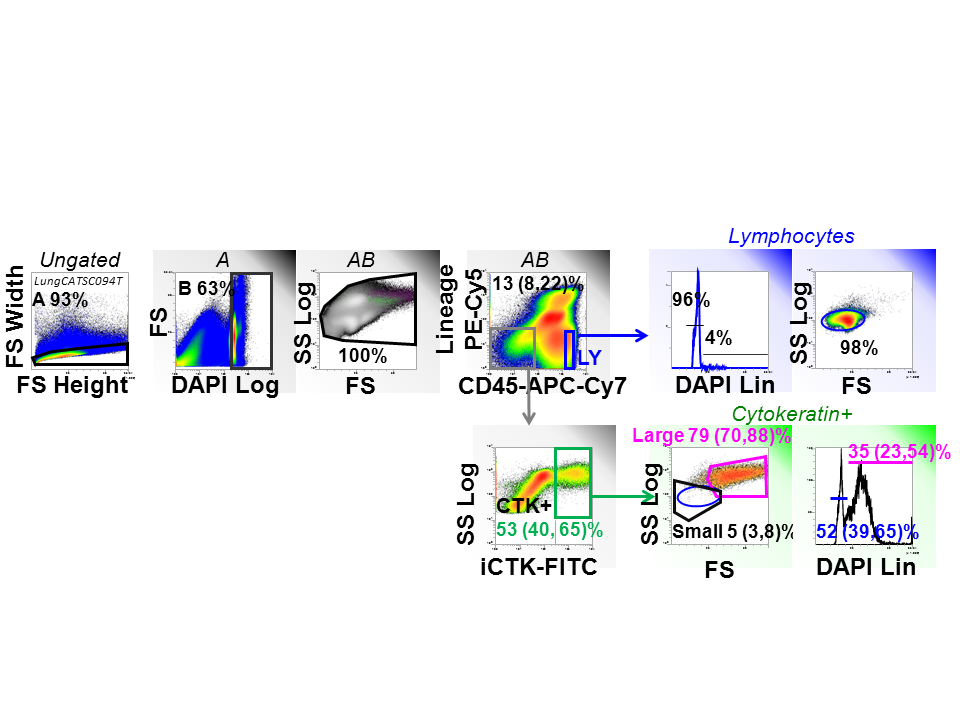

Supplement: Figure S1 — Gating strategy. The gating strategy used to remove cell clusters, debris, red and white blood cells and mesothelial cells is shown. A freshly excised non-small cell lung carcinoma was disaggregated and prepared for flow cytometry as described. 4.9 million events were acquired. Region percents indicate mean values (lower and upper 95% confidence intervals) for 21 NSCLC tumors. Top panels (left to right): Forward light scatter pulse analysis is used to eliminate cell clusters and retain singlet cells (A); the DNA stain DAPI is used to define nucleated cells with ≥2N DNA (B); Forward versus side light scatter is shown for total nucleated cells; CD45 versus a cocktail of CD14, CD33 and glycophorin A is used to gate out hematopoietic (heme) cells; CD14+ is also expressed on mesothelial cells which may be present in pleural effusions; Tissue lymphocytes, identified as CD45bright, are used as an internal standard for 2N DNA content and low light scatter morphology. Bottom panels (left to right): Detection of cytokeratin+ cells on non-heme singlets with ≥2N DNA; enumeration of small (lymphoid light scatter) cells among cytokeratin+ cells; DNA content of cytokeratin+ cells (2N and >2N). This gating strategy was used for all subsequent analyses. (TIF) [file pone.0052885.s001.tif]

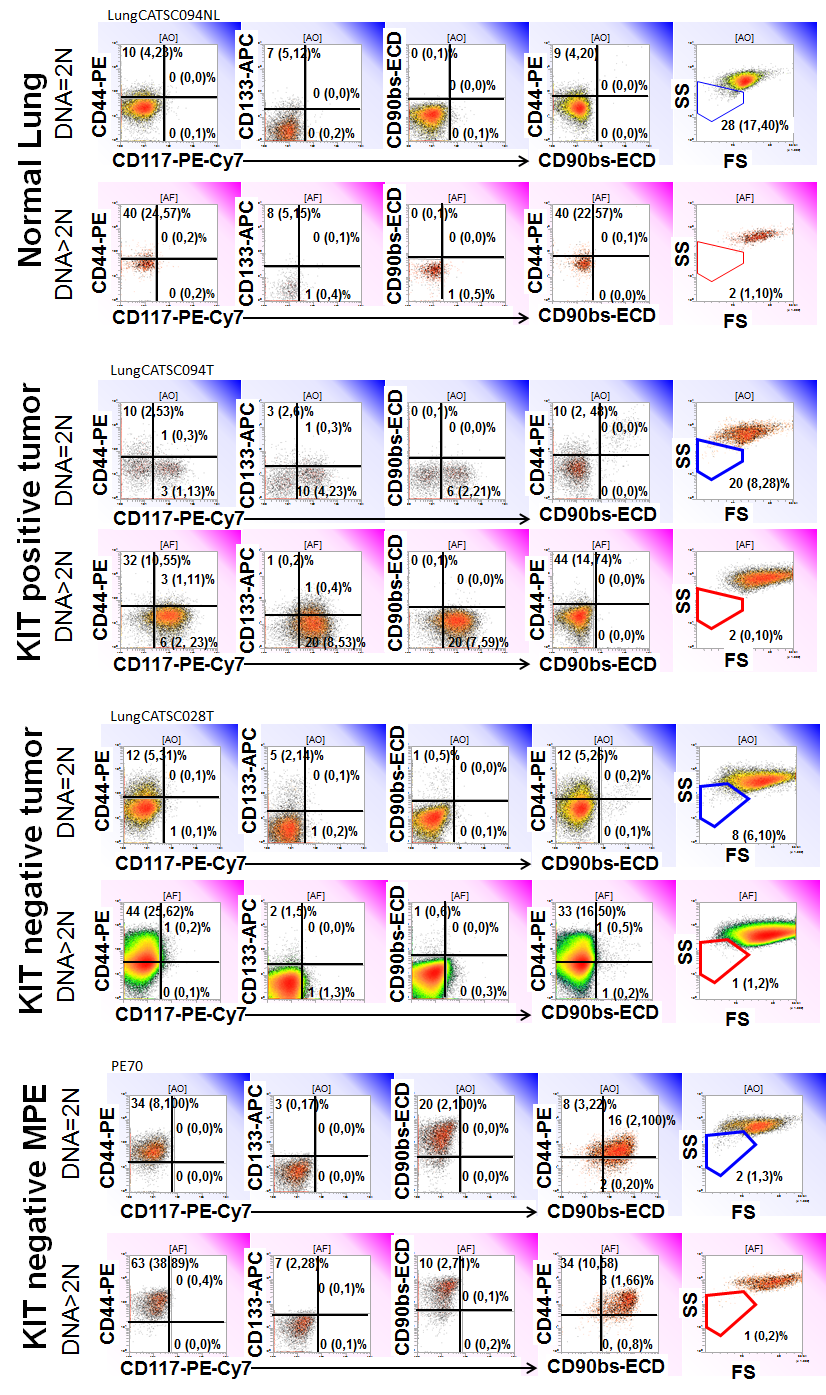

Supplement: Figure S2 — Flow cytometric detection of stem/progenitor markers on cytokeratin+ cells. The gating strategy described in supplementary figure S1 was used to eliminate sources of artifact and identify cytokeratin+ cells. To identify cycling/aneuploid cells, cytokeratin+ cells were divided into those with 2N DNA and those with >2N DNA (Figure S1). Representative analyses are shown for normal lung, KIT positive and negative tumors, and a metastatic pleural effusion. The numbers shown in the analytical regions represent the geometric means (percent of 2N or >2N cytokeratin+ cells) of determinations made on all samples. Parentheses enclose the lower and upper 95% confidence intervals about the geometric means. (TIF) [file pone.0052885.s002.tif]

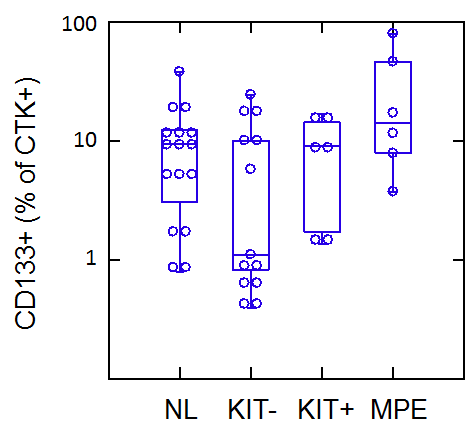

Supplement: Figure S3 — Expression of CD133 on cytokeratin+ cells. (TIF) [file pone.0052885.s003.tif]

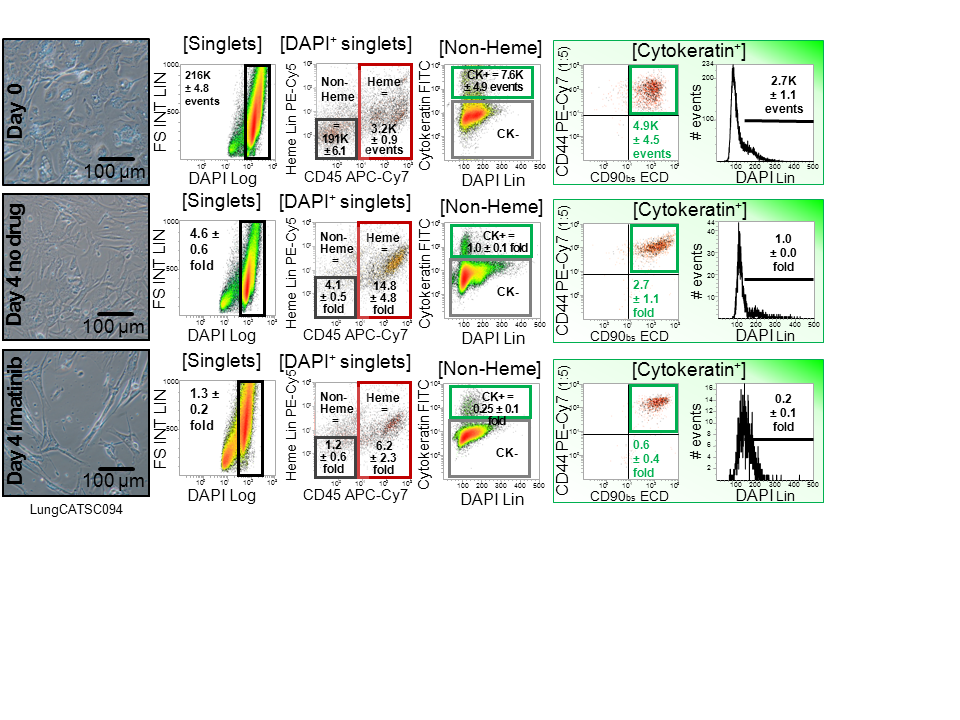

Supplement: Figure S4 — Effect of imatinib on primary lung cancer epithelial cultures. First passage tumor cell isolates were cultured for 4 days in the absence or presence of imatinib (5 μM). Cells were gently trypsinized, counted and analyzed by multi-parameter flow cytometry. Three individual CD117+ tumor preparations were analyzed in duplicate. Photomicrographs and histograms are from a single representative experiment. Event numbers and fold-changes were calculated from the pooled data of 3 independent experiments (mean ± standard error). Singlet cells with ≥2N DNA (first histogram column) increased 4.6-fold in culture, but did not significantly proliferate in the presence of drug. CD45-/heme-lineage- cells also increased 4-fold in culture in the absence, but not the presence of drug, while CD45+ inflammatory cells increased in number in the absence (14.8-fold) or presence (6.2-fold) of drug. Although imatinib caused stasis of non-hematologic cytokeratin- stromal cells, it actually decreased the number cytokeratin+ cells 0.2-fold) and its CD90+/CD44+ subset, a phenotype associated with tumor stem/progenitor cells. The broadening of the cell cycle profile in the presence of imatinib (last column, bottom row) is consistent with drug-mediated cell cycle arrest in S-phase. The bar indicates cells with >2N DNA content (compared to an internal control: resting CD45+ lymphocytes). (TIF) [file pone.0052885.s004.tif]
